# Enigmatic PreS deletions in hepatitis B virus DNA

**DOI:** 10.1007/s11262-020-01805-w

**Published:** 2020-11-05

**Authors:** Wolfram H. Gerlich, Dieter Glebe

**Affiliations:** Institute for Medical Virology, Justus Liebig University Giessen, Giessen, Germany

In this issue of Virus Genes, *Ting Wang* et al. describe a defective variant of hepatitis B virus (HBV) with a very high replication capacity comprising a large deletion in the preS1 domain of the open reading frame encoding the three (large, middle, and small) HBV surface proteins (LHBs, MHBs, and SHBs) [1]. The variant was isolated and cloned from the serum of a patient suffering from chronic hepatitis B (CHB) who had experienced an HBV breakthrough under antiviral therapy with the nucleotide analog adefovir.

The variant showed the typical mutations A181T and N236T in the reverse transcriptase domain of the viral polymerase protein leading to adefovir resistance after previously failed lamivudine therapy. The first lamivudine-selected mutation (A181T) happens to generate the stop mutation W172* in the overlapping S domain of the HBs proteins causing carboxyterminal truncation and intracellular retention of all three HBs proteins. While this is an additional stop mutation in the S domain (C69*) that prevented the formation and release of enveloped HBV particles encoded by the variant, HBV DNA and HBsAg of the variant could nevertheless be detected in the patient serum. This indicated that the patient’s hepatocytes replicating the variant via circular covalently closed (ccc) HBV DNA contained HBV genes encoding functional HBs proteins as well, most likely as linear-integrated HBV DNA fragments. Transcomplementation with functional HBs genes of defective ccc DNA has recently been shown in CHB patients by Peiffer et al. [2].

Surprisingly, the variant described in [1] with the 68 amino acid long preS1 deletion replicated HBV DNA much more efficiently than wildtype (WT) HBV DNA of the same HBV subgenotype C2 when transfected into the HBV permissive hepatoma cell line Huh7. The variant contained also two mutations in the core promoter (A1762T/G1764A) which are known to enhance HBV replication since long [3], but the authors of ref [1] could elegantly prove that the major effect on replication came from the preS1 deletion. When the deleted sequence was artificially re-introduced into the variant, the replication decreased to levels comparable to WT. In addition to enhanced replication, the deletion variant showed a profoundly changed intracellular localization of the HBV core (HBc) protein from predominantly cytoplasmic to almost exclusively nuclear although the HBc protein was unchanged.

Both effects are probably linked together. The preS1 sequence (aa 42-110) deleted in the variant of [1] includes the binding site (aa 103-124) of the LHBs protein to mature HBc particles containing replicated HBV DNA. This binding initiates envelopment of HBc particles and secretion of mature HBV particles [4]. The naked HBc particles remain initially cytoplasmic but can be subject to nuclear import as has been shown for infectious HBV after removal of the HBs envelope [5]. After nuclear entry, the partially double-stranded virion HBV DNA is released from HBc particles, converted to cccDNA, and can enter new replication cycles while the core protein can re-assemble to empty nonfunctional HBc particles [5]. This leads to the phenotype which is described in [1].

At the first glance, the variant may appear irrelevant because it cannot generate infectious HBV progeny. However, the large amounts of partially double-stranded HBV DNA accumulating in the nucleus in absence of functional HBs proteins are subject to potentially detrimental cellular DNA repair processes including integration of HBV DNA fragments. Thus, mutated HBs proteins may directly contribute to pathogenesis of HBV-related diseases like hepatocellular carcinoma (HCC) via the effect of intranuclear accumulation of HBV DNA. In fact, HBV variants with altered HBs structure including preS deletions have increasingly been identified in patients with active HBV disease including HCC [6]. Besides the effect of the enhanced HBV DNA replication, the nonsecreted HBs proteins of the variant may contribute directly to pathogenesis and oncogenicity. WT HBV expresses the preS2 domain as part of MHBs and LHBs which assemble with an excess of SHBs protein to HBV and subviral HBsAg particles ready for secretion. Disturbance of assembly and blocked subsequent secretion occurs with C-terminally truncated HBs proteins or in the absence of WT SHBs as in the case of the variant described in ref [1].

Already decades ago, secretion-defective HBs mutants, e.g., a truncated form of the middle HBs protein MHBs^t^, have been linked to the development of hepatocellular carcinoma (HCC) [7]. Accumulation of HBs proteins causes ER stress and may contribute to development of HCC [8]. However, ER stress may not be the only or main oncogenic mechanism of mutated HBV variants. The preS2 domain is known to carry a small transcription transactivating domain if present in the cytosol, may it be part of MHBs^t^ [7] or of WT LHBs [9]. The preS2 domain of LHBs or MHBs could still be expressed by the deletion variant of ref [1]. Although ref [1] did not study this aspect or oncogenicity of the variant, other studies [10, 11] have shown that preS2 epitopes can be very often detected in HBV-related HCC tissue and a partial MHBs protein covering the entire preS2 sequence was shown to transactivate oncogenesis supporting genes, e.g., hTERT [10].

Seemingly contrary to the published association of preS2 with HCC [10, 11], a high prevalence of preS deletions, both in preS1 and/or preS2, has recently been reported in sera of HCC patients [12]. Remarkably, patients who experienced recurrence of the HCC after surgical removal had a significantly higher proportion of HBV DNA with certain combinations of preS deletions than patients without recurrence [12]. Ref [12] provides no hints on the mechanisms inducing oncogenesis by the various deletions, but an enhanced replication of HBV DNA and its nuclear accumulation as shown in ref [1] would be plausible candidates among many others.

The fact that the highly replicating HBV variant of ref [1] was found in a patient after failed adefovir therapy following lamivudine resistance illustrates the potentially detrimental results of suboptimal antiviral HBV drug therapies facilitating inaccurate viral replication and generation of defective HBV genomes with enhanced pathogenicity.
